# Diversity and composition of farm plantation tree/shrub species along altitudinal gradients in North-eastern Ethiopia: implication for conservation

**DOI:** 10.1016/j.heliyon.2022.e09048

**Published:** 2022-03-05

**Authors:** Meseret Muche, Eyayu Molla, Boris Rewald, Berhanu Abraha Tsegay

**Affiliations:** a Department of Biology, Woldia University, P.O. Box, 400, Woldia, Ethiopia; b Department of Natural Resource Management, College of Agriculture and Environmental Sciences, Bahir Dar University, Bahir Dar, Ethiopia; c Institute of Forest Ecology, Department of Forest and Soil Sciences, University of Natural Resources and Life Sciences Vienna, Austria; d Department of Biology, Bahir Dar University, Bahir Dar, Ethiopia

**Keywords:** Agroforestry, Conservation, Farmers acuity, Plantation niches, Species diversity

## Abstract

On-farm tree plantation is a form of land use where trees are planted at the edge or interspersed with crops. It has been practiced in different parts of Ethiopia due to its contribution to the household economy and soil fertility. This study was carried out to evaluate the variation in tree/shrub plantations along altitudinal gradients and plantation niches, and farmers' on-farm tree plantation practices at Kobo and Guba Lafto districts, North-eastern Ethiopia. Transect walks and semi-structured questionnaire were administered to appraise farmers' tree/shrub plantation practices and compositions between August and December 2020. A total of 135 plots along altitudinal gradients (Forty-five sample plots per altitude) and 135 retrieved questionnaires (45 per altitude) were analyzed. At each plot, tree/shrub richness, diversity, stem density, and important value index (IVI) were computed. Multivariate analysis, descriptive statistics, and preference rankings were used to evaluate vegetation data and farmers’ perceptions on tree/shrub plantations. The results showed that most farmers (78.5 %) integrate trees with their crops for household use and soil fertility maintenance. The multivariate analysis revealed a significant reduction in the number of taxa, stem density, richness, and diversity with increasing elevation, from homestead to the boundary and on-farm plantation niches. *Ziziphus spina-christi* and *Cordia africana* were the most preferred tree species; Fabaceae was the dominant family representing 18.9 % of the species. The results emphasized considerable variations in relative density, relative dominance, and important value index (IVI) across altitudinal gradients and plantation niches. *Acacia seyal* and *Z. spina-christi* contributed the highest IVI at lower and middle elevations, whereas *Eucalyptus globulus* had high IVI at a higher elevation. In the study districts, the distribution of multifunctional indigenous tree plantations gradually decreases with the entire altitudinal gradients compared to exotic trees/shrubs. This calls for substantial efforts on the propagation and conservation of native tree and shrub genetic resources.

## 1 Introduction

The world population is expected to exceed 9.3 billion by the mid-century, and thus, the demand for more productive land needs urgent attention (FAO, 2020). Many African countries have continued to experience food insecurity, decline in per capita farm income, soil degradation, and aggravated biodiversity loss (Vlek et al., 2010). In Ethiopia, population pressure has resulted in increasing demand for lands for food, energy, and other resources (Amsalu et al., 2007) leading to the conversion of forests and grazing lands into croplands (Wondie et al., 2011). In the wake of deforestation and degradation of natural vegetation and associated negative impacts on natural resources, the government of Ethiopia has launched an initiative to foster sustainable soil management strategies (Abebe, 2005; Wondie and Mekuria, 2018). In particular, agroforestry (AF) has been identified as part of the solution to address the decline in soil fertility and deforestation (Abebe et al., 2010; FAO, 2020). AF is a dynamic and complex ecological-based natural resource management system where farmers intentionally retain or integrate trees into their farmland in various spatio-temporal arrangements (Nair, 1998; Dhakal et al., 2012; Bucagu et al., 2013). AF systems are the main reservoirs of biodiversity and provide other ecosystem services, such as reduced soil erosion, enhanced carbon sequestration, holding high mitigation, and adaptation potentials under progressing climate change (Pandey et al., 2016; Reang et al., 2021). AF systems can be considered an important component of the (regional) reducing emissions due to deforestation and forest degradation (REDD+) strategy while simultaneously sustaining the livelihood of the rural population (Reang et al., 2021).

A complex of factors determine the composition of AF systems (Nogues-Bravo et al., 2008; Dhakal et al., 2012; Solefack et al., 2018; Reang et al., 2021). Among other factors, topographic gradients (slopes, elevation, and aspects), plantation niches, land use management, cultural diversity, and varying-rainfall pattern have been found to affect the functional composition of on-farm tree species (Costa et al., 2017; Pandey et al., 2021; Reang et al., 2021). Notably, elevation and tree plantation niches, such as home gardens, parklands, coffee shade tree systems, scattered trees on farmland, and boundary plantations are the principal factors that considerably affect the distribution of plant species (Molla and Kewessa, 2015; Legesse and Negash, 2021; Pandey et al., 2021). An increasing elevation is usually related to a lower temperature and higher humidity and hence this climatic variability strongly shapes the composition of vegetation in general and trees used for AF purposes in particular (Nogues-Bravo et al., 2008; Solefack et al., 2018).

In Ethiopia, a common AF system integrates *Ensete ventricosum* and *Coffea arabica* as scattered trees on cultivated lands in association with cereal crops such as *Zea mays*, *Eragrostis tef*, *Sorghum bicolor*, etc (Duguma and Hager, 2010; Kebebew and Urgessa, 2011). Farmers are also frequently integrating fruit crops such as *Persea americana* Mill., *Mangifera indica* Wall., *Psidium guajava* L., *Casimiroa edulis* S. Watson, *Ananas comosus* (L.) Merr., and *Musa* spp., in their agricultural lands (Negash and Starr, 2015) for food, income, shade, and soil fertility improvements. These tree/shrub plantations in agricultural systems are increasingly promoted as facilitating economic and socio-cultural services, biodiversity conservation, and an array of other ecosystem services benefitting smallholder farmers and rural communities (Nair, 1998; Kebebew and Urgessa, 2011; Abebe et al., 2013). The ecosystem services provided by plantations include rehabilitating degraded lands (Bishaw et al., 2013), thermal comfort and/or shading (Pinho et al., 2012), alleviating temporal shortages of water and energy, and facilitating adaptation to climate change (Bishaw et al., 2013; Coe et al., 2014). Thangataa and Hildebrand (2012) and Mbow et al. (2014) asserted that the inclusion of trees in agricultural systems can optimize nutrient cycling and impart a positive effect on soil physicochemical properties. For instance, tree-based AF may increase the soil potassium content three times over croplands without tree integration (Ulery et al., 1995). According to Drechsel et al. (1991), *Cassia siamea* and *Azadirachta indica* were superior in enriching the sandy-loamy topsoil with calcium and increasing soil pH in central Togo. In Zambia, Yengwe et al. (2018) reported an increase in nitrogen by 18 kg ha^−1^ year^−1,^ and microbial diversity and abundance, by litter inputs of *Faidherbia albida* intercropped with maize. In addition to positive effects on the nutrient cycle, the inclusion of trees within croplands may increase soil organic carbon (SOC) stocks and soil water infiltration rates (Sanou et al., 2010; Chatterjee et al., 2019).

In Ethiopia, few studies have been conducted on the Spatio-temporal variation in crop diversity within AF systems, the practices and benefits of increasing tree diversity in farmed landscapes, and carbon stocks in AF systems (Abebe et al., 2010; Endale et al., 2017; Amare et al., 2019; Birhan and Abebe, 2019; Derero et al., 2020; Betemariyam et al., 2020). Yet, further studies need to be conducted on tree species composition along altitudinal gradients and plantation niches on-farm systems in North-eastern Ethiopia. And thus, the present study was motivated by the fact that exploration of tree plantation practices based on elevation and plantation niches are essentially required for the implementation of conservation and propagation actions of multi-purpose tree species especially crucial for soil fertility improvement. Therefore, the objectives of this study were: (1) to compare differences in species richness, diversity, stem density, and IVI of plantation trees/shrubs on farmlands along with altitudinal gradients and plantation niches; and (2) to assess farmers' perception of AF systems and its role on the maintenance of soil fertility.

## 2 Methods

### 2.1 Study sites description

**Figure 1:**
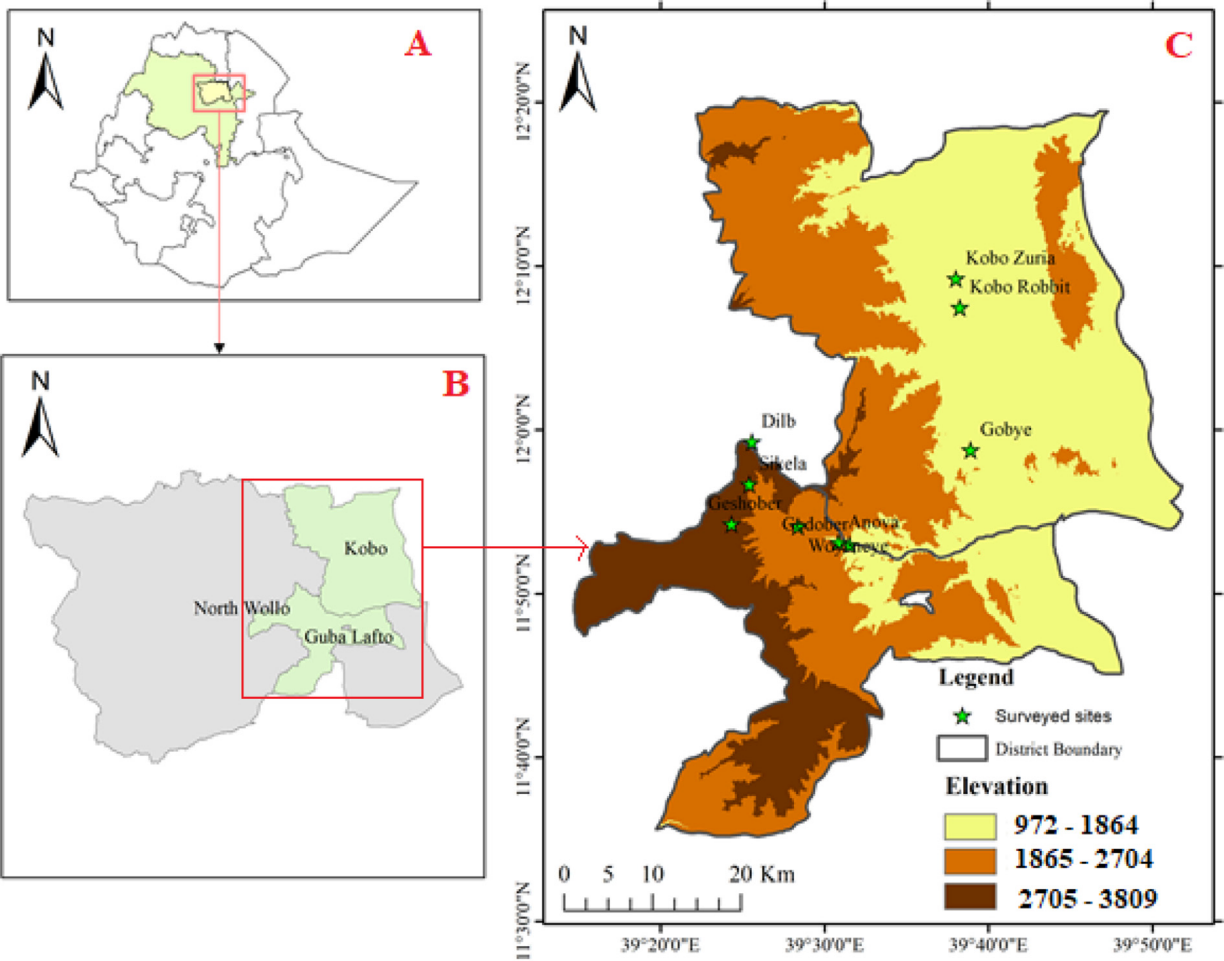
Geographical Location of the study area (A) political map of Ethiopia by regions (B) North Wollo administrative zone of the Amhara regional state by study districts (Guba Lafto and Kobo) and (C) the study sites (9) based on altitudinal gradient.

The study was conducted at nine sites along an elevational gradient stretching between two districts: Kobo (11°51'45.63" to 12°19'24.97"N and 39°19'54.87" to 39°53'2.33"E) and Guba Lafto (11°34’54’’to 11°58’59’’N and 39°6’9” to 39°45’58’’E), both located in the North Wollo Administrative Zone, North-eastern Ethiopia (Figure 1, Supplementary 1). The Kobo district (lowland) is characterized by fertile, plain land (65 %) while 20, 11, and 4 % are hillock landforms, rugged, and gorges, respectively, with an altitudinal range between 972 to 1864 m a.s.l (NWZAD, 2019). In contrast, the Guba Lafto district (mid-altitude lands to highland) holds gorges (15 %), mountainous/hills (35%), escarpments (30 %), and plateau (20 %) terrains with wide relief differences ranging from mid-altitude (1865–2704 m a.s.l.) to highland (2705–3809 m a.s.l.) (NWZAD, 2019, Figure 1). Ten-year climatic data showed that the Kobo district is particularly prone to drought with an erratic unimodal rainfall pattern; holding mean monthly precipitation ranging from 3.3 mm in January to 199.2 mm in August, and an average annual rainfall of 50 mm (ARKWF, 2020). Kobo district features a uniform high temperature throughout the year, with average minimum and maximum temperatures of 15.1 °C and 30.7 °C, respectively (ARKWF, 2020). The Guba Lafto district holds a bimodal rainfall pattern with an erratic distribution in precipitation varying widely across the district and years (NWZAD, 2019). In the midland of the Guba Lafto district, the mean monthly rainfall ranges from 10.8 mm in January to 380.1 mm in August and the average annual rainfall is 98.3 mm. The mean monthly temperature ranges between 12.4 °C and 28.7 °C (ARKWF, 2020). At highlands, the mean monthly rainfall ranges from 47.7 mm in January to 600 mm in August with an average monthly rainfall of 320.2 mm. The mean monthly temperature at highlands ranges between 8.9 °C and 22.7 °C. In the studied districts, the plots on steeper slopes are dominantly covered by shallow soils, mainly Leptosols, Regosols, Fluvisols, and Andosols; the plateaus are covered by clay soils that can be described as Vertisols and Vertic Cambisols (FAO, 1999). Cereals (*Zea mays, Sorghum bicolor*, *Eragrostis tef*) and pulses (*Cicer arietinum*, *Pisum sativum*, etc.) are the major crop types grown in the study districts, alongside fruit trees such as *Mangifera indica*, *Persea americana*, *Carica papaya*, etc.

### 2.2 Data collection on on-farm tree and shrub species

**Figure 2:**
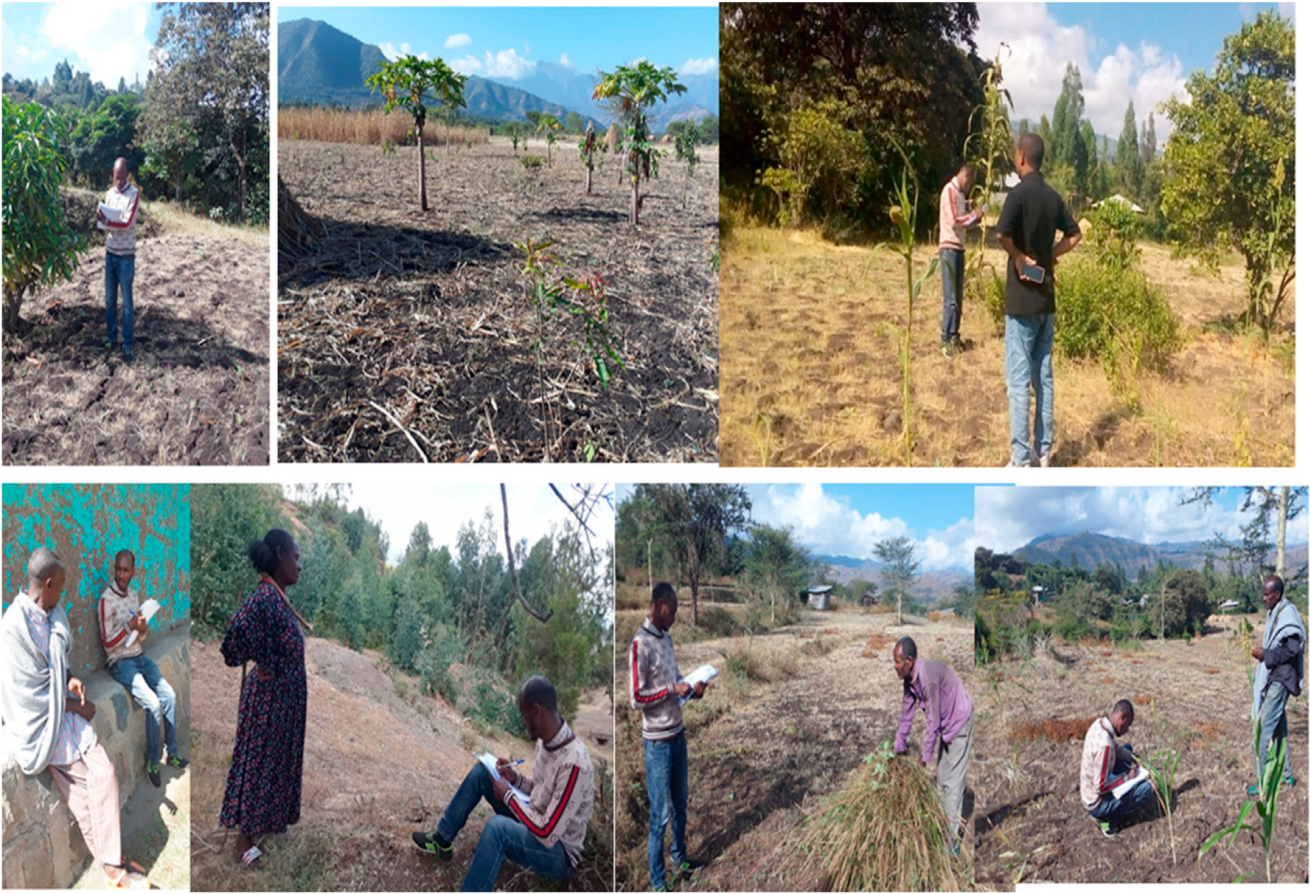
An illustration on the tree/shrub plantation inventory and interviewed the farmers on their perception of plantation practices on-farm systems (Photo by Meseret Muche, 2020).

In August 2020, a survey was conducted to get a general overview of the tree plantation practices on-farm systems. Transect walks were used to collect data on the tree/shrub plantation compositions and diversity in the selected areas using a two-stage sampling approach. Primarily, Kobo (lowland) and Guba Lafto (midland and highland) districts were purposely selected based on their tree plantation practices on farmlands, elevation gradients, and plantation niches (on-farm, boundary planting, and homestead plantation) (Figure 1; Figure 2). Then, three sites (i.e. villages) at each altitude were selected using a simple random sampling technique to assess the farmers' plantation practices and woody vegetation composition of agroforestry (AF) systems (Figure 1; Supplementary 1). During transect walks, trees and shrub species and integrated crops were recorded for a quantitative vegetation data inventory, using pre-prepared field observation data collecting tools (such as the number of tree/shrub taxa (Taxa-S), stem density, richness (R), diversity (H'), relative frequency (RF), relative density (RD), relative dominancy (RDo), and Important Value Index (IVI) along the elevation gradient and plantation niches). A total of 135 sample plots (15 plots per site, 45 plots at each altitudinal level) were surveyed for the study. Ecological indices characterizing the tree and shrub vegetation in the AF systems were computed using the formulas in Table 1. At the end of vegetation data inventories, samples of tree/shrub species with their local names were collected across altitudinal gradients and niches, pressed, and dried for species identification. The scientific names were identified and verified based on the Natural Database for Africa (NDA) version 2.0 (http://alnapnetwork.com/NDA.aspx), International Plant Name Index (https://ipni.org), and published Floras of Ethiopia and Eritrea (Hedberg and Edwards, 1989; Edwards et al., 1995, 1997; Hedberg et al., 2003).

**Table 1:** Indices characterizing trees and shrubs in agroforestry systems in North-eastern Ethiopia by altitude (n = 3), tree niches (n = 3), and study sites (n = 9).

Ecological indexes		Equation	References
R	Menhinick's	$\text{R ​}=\text{ ​}\text{S}/\sqrt{\text{N}}$	Rejmánek and Randall (1994)
H'	Shannon-Wiener	${\text{H}}^{\prime }=-\sum_{i=1}^{s}Pi\;\ln\;Pi$	Kent and Coker (1992)
SD	Number of a tree/shrub species	$\text{SD ​}=\text{ ​}\frac{\text{Number ​of ​a ​species}}{\text{Total ​area ​sampled}}$	Kent and Coker (1992)
RF	Number of species	$\text{RF ​}=\text{ ​}\frac{Numbered\;of\;occurance\;of\;the\;spp}{Numbered\;of\;occurance\;of\;all\;spp}*100$	Magurran (1988)
RD	Numerical strength	$\text{D ​}=\text{ ​}\frac{Density\;of\;the\;spp}{Density\;of\;all\;spp}*100$	Magurran (1988)
RDo	Species abundance	$\text{RDo ​}=\text{ ​}\frac{Total\;No\;of\;spp\;encountered}{Number\;of\;spp\;occurance}*100$	Magurran (1988)
IVI	Importance of species	IVI = RF + RD + RDo	Kent and Coker (1992)

R, Richness; H', Shannon Diversity Index; SD, Stem Density; RF, Relative Frequency; RD, Relative Density; RDo, Relative Dominancy; IVI, Important Value Index; N = the number of tree species; S = the number of species; Pi = the proportion of individuals of the i^th^ species expressed as a proportion of total cover in the sample, and ln = the natural logarithm.

### 2.3 Questionnaire survey

Farmer respondents that are practicing tree/shrub plantations on their farmlands were selected at each altitudinal level using a snowball sampling approach. At each elevation level, 45 farmers were selected to administer a semi-structured questionnaire (Supplementary 2), so that a total of 135 farmer informants from different gender and age groups were used for this study. Both the interviews and vegetation data inventory (see above) were carried out simultaneously between August and December 2020. Additionally, ten key informants were questioned to rank the most preferred tree species used for soil fertility maintenance in their farmlands. The key informants were chosen based on traditional knowledge of farmland plantation practices following the suggestion made by the districts developmental association (DAs). The interview questionnaire included the socio-demographic variables (gender, age, educational and marital status, and family size), farmers' perception of the inclusion of tree/shrub plantation on agricultural lands, and their tree preferences to maintain soil fertility (Supplementary 2). It was administered using the local language, Amharic, and later translated into English. The interview was supplemented by direct observation and transects walks (see above). Furthermore, the study was approved by the research and ethical review board of Woldia University Faculty of Natural and Computational Science with the reference number FNCS 0008/2014. Moreover, all respondents were aware of the purpose of the study and consented to participate in the survey.

### 2.4 Statistical analyses

The effects of the altitudinal gradient (lowland, mid-altitude, and highland), tree and shrub plantation niches (on-farm/farmland, homestead, and boundary) and their interactive effects on the number of Taxa (Taxa-S), stem density, species richness (R), diversity (H’), relative frequency (RF) and density (RD), Important Value Index (IVI), and relative dominance (RDo; Table 1) were analyzed by a General Linear Model (GLM) using SPSS v.24 and PAST v. 3.04 software packages (Hammer et al., 2001). Posthoc comparisons of means were employed using Least Significant Difference (LSD) at p < 0.05. Furthermore, preference ranking and descriptive statistics were used to evaluate the farmers’/experts perception of the benefits of AF in general and tree species preferences regarding soil fertility maintenances in specific.

## 3 Results

### 3.1 Background information on the characteristics of the respondents in the surveyed districts

The socio-demographic information in Table 2 revealed that among the surveyed households there were more male respondents 102 (75.56 %) compared to females 33 (24.44%). The mean age was 48.4 years, in which minimum and maximum ages were 18 and 75 years, respectively. The fact that age was an important variable could tell the farmers' experiences and knowledge in the identification of major tree and shrub plantations on agricultural systems and the implication for soil fertility maintenance. Concerning education status, 57 (42.22 %) of the respondents had no formal education, while 53 (39.26 %) had primary and secondary schoolings, and 25 (18.52 %) had some schooling in tertiary education. Most of the surveyed respondents 111 (82.2 %) were married, and only 5 (3.7 %) of the individuals were divorced or widowed. The survey results also showed that the average family size was 6, ranging from 0 to 11 (Table 2). The land is the major asset of the farmers in the study areas to guarantee sustainability and implement the AF practices and thus most of the surveyed farmers (43.7 %) own less than 0.5 ha of land. They further reported that the presence of small-sized farmland is the major problem in practicing AF in the farm systems (Table 2).

**Table 2:** Socio-demographic characteristics of sampled households (n = 135) at nine study sites (45 per elevation).

Household Characteristics		Frequency	Percent
Gender	Male	102	75.56
	Female	33	24.44
Age (yrs)	18–30	29	21.48
	31–40	35	25.93
	41–50	30	22.22
	>50	41	30.37
Educational status	Tertiary	25	18.52
	Primary & secondary	53	39.26
	No formal schoolings	57	42.22
Marital status	Married	111	82.2
	Unmarried	19	14.1
	Others	5	3.7
Family size	1–5	69	51.1
	6–10	57	42.2
	>10	9	6.7
Landholding (ha)	<0.5	59	43.7
	0.5–1	53	39.3
	>1	23	17.0

### 3.2 Effect of altitudinal gradients and plantation niches on tree/shrub composition

#### 3.2.1 Tree species richness and diversity on agricultural lands

**Figure 3:**
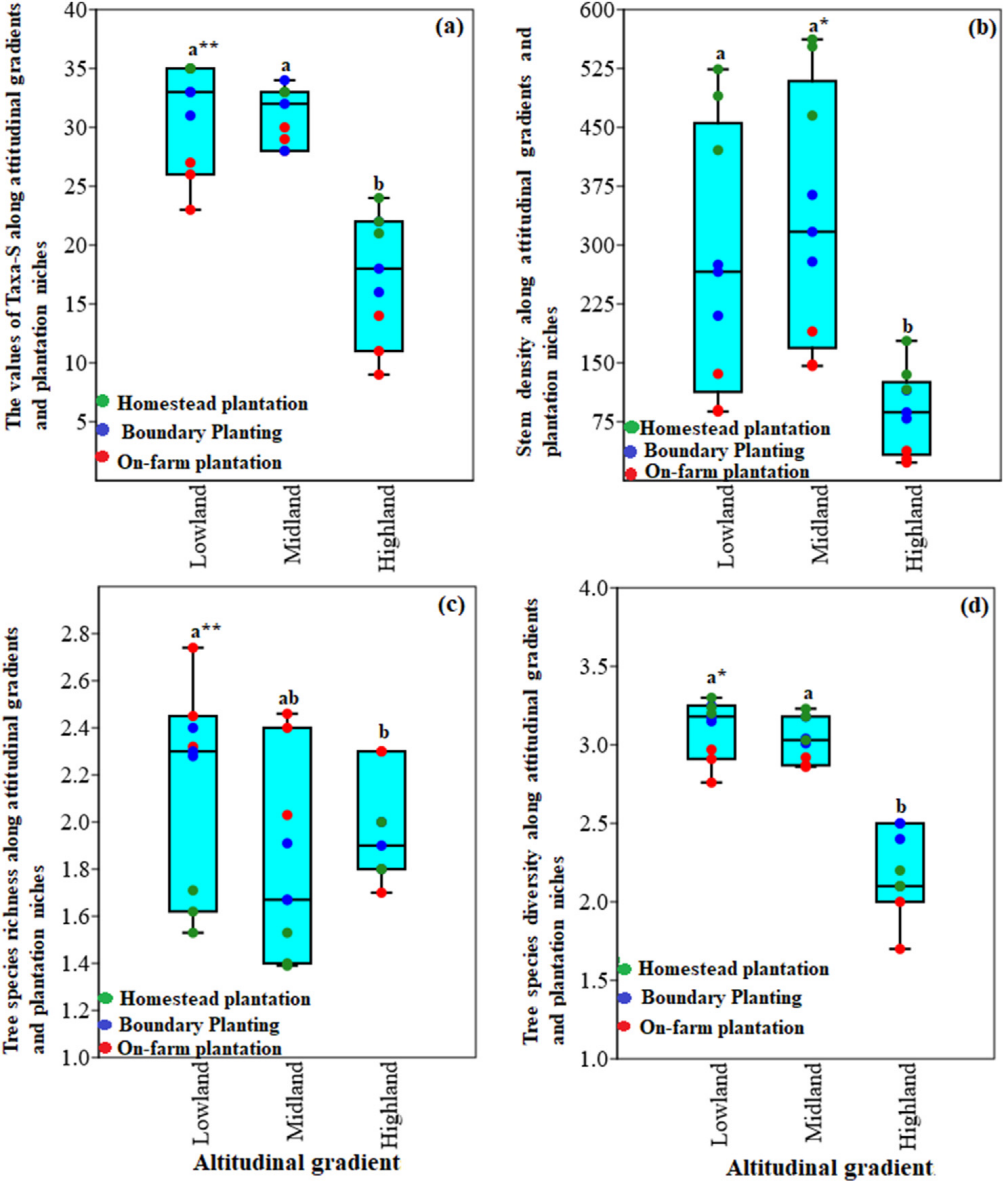
Effect of an altitudinal gradient and on-farm tree plantation niches (i.e. Homestead plantation, Boundary planting, on-farm planting) on the (a) number of taxa (Taxa-S), (b) stem density, (c) richness (R), and (d) Shannon diversity (H’) in agroforestry systems of North-eastern Ethiopia. Different letters denote significant differences between means across altitudinal gradient.

The numbers of taxa (Taxa-S), stem density, richness, and diversity of tree plantation in agroforestry systems of North-eastern Ethiopia, as related to elevation and tree niches, are presented in Figure 3. Both numbers of woody taxa and stem density showed a statistically significant reduction along the altitudinal gradient (Table 3), in both cases driven by the lower numbers of taxa (11.3 ± 2.5) and stem densities (29.7 ± 7.6) in highland AF systems (Figure 3). Both parameters varied according to tree niches, with generally greatest numbers of taxa and stem densities at homesteads, followed by boundary plantings and least values of woody plants on-farm (p < 0.001); however, numbers of taxa on mid-elevation plots possess in general less variability and no clear separation according to planting niches (Figure 3a). Both greater numbers of woody taxa and stem densities of on-farm plantations and partially boundary plantings at mid-elevation plots compared to lowland plots seem to drive the significant interaction effects between altitudes and niches (Table 3). Regardless of these variations, *A. seyal* and *Z. spina-christi* gained dominance at the low and mid altitudes while *E. globulus* dominated the highland farms. Concerning the gross numbers of tree plantations on-farm systems, a total of 37 species belonging to 20 families were recorded across elevations. Fabaceae was the most dominant family, represented by 7 (18.9 %) species, followed by Anacardiaceae, Boraginaceae, Euphorbiaceae, Myrtaceae, and Rutaceae (represented by 3 (8.1 %) species each) (Supplementary 3). The result highlighted that tree species richness was statistically (p < 0.01) greater in the lower elevation plots, followed by mid and the highlands, which also had considerable differences (p < 0.01) across plantation niches (Figure 3c; Table 3). Similarly, tree species diversity (H’) was significantly (p < 0.001) lower at highlands AF systems (H’ = 1.9 ± 0.2), with a markable lower diversity on-farm compared to homesteads at low and mid-elevation plots while at highland plots the greatest diversity was found among boundary plantings—likely underlying the significant interaction effect (Figure 3d; Table 3).

**Table 3:** General Linear Model results on the effect of altitude, tree/shrub plantation niches and their interactive effects on variables related to woody species assemblage in agroforestry systems of North-eastern Ethiopia. See text for details on variables.

Variable	Effect	DF	F	P
Taxa-S	Altitudinal Gradients (AG)	2	145.9	.000
	Tree Niches (TN)	2	41.9	.000
	AG x TN	4	2.99	.047
Stem density	Altitudinal Gradients (AG)	2	118.1	.000
	Tree Niches (TN)	2	143.5	.000
	AG x TN	4	13.4	.000
Richness	Altitudinal Gradients (AG)	2	7.5	.004
	Tree Niches (TN)	2	31.2	.000
	AG x TN	4	4.7	.009
Diversity	Altitudinal Gradients (AG)	2	248.9	.000
	Tree Niches (TN)	2	31.3	.000
	AG x TN	4	4.6	.010
RF	Altitudinal Gradients (AG)	2	166.9	.000
	Tree Niches (TN)	2	0.2	.81
	AG x TN	4	0.1	0.9
RD	Altitudinal Gradients (AG)	2	27.2	.000
	Tree Niches (TN)	2	19.9	.000
	AG x TN	4	18.6	.000
RDo	Altitudinal Gradients (AG)	2	206.1	.000
	Tree Niches (TN)	2	39.9	.000
	AG x TN	4	39.9	.000
IVI	Altitudinal Gradients (AG)	2	247.2	.000
	Tree Niches (TN)	2	23.2	.000
	AG x TN	4	23.7	.000

The total sum of squares (SS) is the same for all models (4, 26), and effects are thus directly comparable across models. RF, Relative Frequency; RD, relative density; RDo, Relative Dominancy; IVI, Important Value Index; DF, Degree of Freedom; F, Fisher test; P, Probability Level.

#### 3.2.2 Tree plantation occurrences and composition on agricultural lands

The proportion of tree species frequencies, density, and dominance on-farm systems based on elevations are given in Tables 3 and 4. Variation in species, relative tree density (RD), and relative dominance (RDo) were observed among the studied altitudinal gradients within tree plantation niches (p < 0.001). Elevation and types of plantation niches are the main predictors that determine the changes in the RD and RDo of species. Thus, the RD and RDo of tree species are greatly distributed in homestead plantations, followed by boundary planting and on-farm agroforestry systems. In addition, there were marked differences in elevation, where there are higher RD and RDo of farm tree assemblage in lowland than in highland. The RD of species ranged from 0.36-10.44, 0.16–10.28, and 0.002–0.15 at low, mid, and high altitudes, respectively (Table 4). *A. seyal* possessed the greatest RD at low and mid-altitude plots, and *E. globulus* at highland plots, whilst *Acacia senegal* and *Dodinia angustifolia* were found to be the lowest in terms of RD. RDo ranged between 1.14-6.12, 1.36–7.03, and 0.007–0.06 at low, mid, and high elevation plots, respectively (Table 4). Consequently, relatively higher species dominance was observed at mid-elevation plots compared to others, of which, *A. seyal* and *Z. spina-christi* were heavily dominating within the intact tree plantation niches (Table 4). However, the relative frequency (RF) of tree plantation was statistically significantly (p < 0.01) different across elevations but did neither show variations within tree plantation niches nor any interaction effects (Table 3). The highest RF was exhibited by *A. seyal* (RF = 5.8; 5.3), *Z. spina-christi* (RF = 5.6; 5.3), and *Psidium guajava* (RF = 4.99; 4.4) at lowlands and mid-elevations, respectively (Table 4). *E. globulus* (RF = 0.12), *Carissa spinarum* (RF = 0.08), and *Ehretia cymosa* (RF = 0.07) had a higher frequency of distribution at highland plots (Table 4). The important value index (IVI) of tree/shrub species was significantly reduced with increasing altitude and within each plantation niche (Tables 3 and 4). The IVI of tree/shrub species ranged from 2.5-22.4, 1.97–22.6, and 0.01–0.32 at low, mid, and high altitudes, respectively. The greatest IVI was recorded for *A. seyal* at low and mid-altitude plots, and for *E. globulus* at highlands. In contrast, *Z. spina-christi*, *Citrus sinensis*, *Rhamnus prinoides*, *Coffea arabica*, and other species had high IVI across the entire altitudinal gradient (Table 4).

**Table 4:** Indicators characterizing woody species (tress/shrubs) assemblages in agroforestry systems along an elevation gradient in North-eastern Ethiopia.

Species	Family	972–1864 m asl (LE)				1865–2704 m asl (ME)				2705–3809 m asl (HE)			
		RF	RD	RDo	IVI	RF	RD	RDo	IVI	RF	RD	RDo	IVI
*Acacia saligna*	Fabaceae	4.9	4.8	3.4	13.2	1.9	1.1	1.9	4.9	0.03	0.02	0.04	0.09
*Acacia senegal*	Fabaceae	0.7	0.4	1.4	2.5	1.8	0.8	1.7	4.3	0.23	0.02	0.04	0.08
*Acacia seyal*	Fabaceae	5.8	10.4	6.1	22.4	5.3	10.3	7.0	22.6	0.02	0.02	0.04	0.08
*Acacia tortilis*	Fabaceae	3.1	2.1	2.3	7.4	3.4	3.3	3.2	9.9	0.03	0.03	0.04	0.10
*Adhatoda schimperiana*	Acanthaceae	1.8	1.3	1.5	4.6	-	-	-	-	-	-	-	-
*Albizia schimperiana*	Fabaceae	1.6	0.9	1.2	3.6	3.6	3.2	3.2	10.0	0.05	0.05	0.05	0.015
*Carica papaya*	Caricaceae	4.7	5.1	3.7	13.6	4.5	5.1	4.0	13.7	0.004	0.003	0.007	0.015
*Carissa spinarum*	Apocynaceae	1.9	1.2	2.1	5.2	2.2	1.1	1.9	5.3	0.08	0.09	0.05	0.23
*Catha edulis*	Celactraceae	1.8	1.4	2.3	5.5	1.9	1.68	3.0	6.6	-	-	-	-
*Citrus aurantifolia*	Rutaceae	2.9	2.1	2.5	7.5	3.2	2.2	2.4	7.9	-	-	-	-
*Citrus medica*	Rutaceae	3.8	3.8	3.4	10.9	5.0	5.5	4.0	14.5	-	-	-	-
*Citrus sinensis*	Rutaceae	3.8	3.5	3.1	10.4	4.7	4.5	3.4	12.7	0.002	0.002	0.016	0.020
*Coffea arabica*	Rubiaceae	2.2	2.3	3.4	8.0	3.3	3.6	3.8	10.7	0.05	0.05	0.05	0.15
*Cordia africana*	Boraginaceae	2.3	1.7	2.5	6.5	3.1	1.72	1.9	6.8	0.04	0.03	0.04	0.11
*Cordia myxa*	Boraginaceae	2.3	1.5	2.3	6.1	1.6	0.8	1.7	4.1	0.04	0.03	0.03	0.1
*Croton macrostachyus*	Euphorbiaceae	3.2	3.4	3.6	10.2	3.6	3.7	3.6	10.9	0.05	0.05	0.04	0.14
*Dodinia angustifolia*	Sapindaceae	3.2	2.3	2.5	8.1	0.4	0.2	1.4	2.0	0.024	0.023	0.023	0.072
*Ehretia cymosa*	Boraginaceae	2.8	2.2	2.7	7.6	2.4	1.4	2.1	5.9	0.07	0.06	0.04	0.17
*Entada abyssinica*	Fabaceae	2.5	2.8	2.4	7.8	3.2	3.8	3.5	10.5	0.06	0.09	0.04	0.19
*Eucalyptus camaldulensis*	Myrtaceae	2.5	3.6	3.0	9.1	2.6	3.0	2.7	8.3	-	-	-	-
*Eucalyptus globulus*	Myrtaceae	-	-	-	-	-	-	-	-	0.12	0.15	0.06	0.32
*Euclea racemosa*	Ebenaceae	1.0	0.5	1.1	2.7	2.9	1.9	2.3	7.2	0.02	0.02	0.02	0.06
*Euphorbia tirucalii*	Euphorbiaceae	2.6	6.1	4.9	13.7	3.2	5.2	5.0	13.4	0.005	0.003	0.007	0.0015
*Ficus vasta*	Moraceae	1.7	1.2	2.4	5.4	1.9	1.0	1.9	4.9	0.02	0.02	0.04	0.08
*Fragaria ​ananassa*	Rosaceae	1.6	0.8	1.7	4.1	2.7	1.7	2.3	6.8	-	-	-	-
*Grevillea robusta*	Proteaceae	2.5	1.9	2.4	6.7	2.8	1.9	2.4	7.1	0.05	0.04	0.04	0.13
*Jatropha curcas*	Euphorbiaceae	1.0	0.7	2.3	4.0	-	-	-	-	-	-	-	-
*Mangifera indica*	Anacardiaceae	3.6	3.2	3.0	9.8	4.8	5.8	4.3	14.9	-	-	-	-
*Moringa stenopetala*	Moringaceae	2.5	1.5	2.1	6.1	1.1	0.6	2.0	3.6	-	-	-	-
*Olea europaea subsp. cuspidate*	Oleaceae	1.4	1.0	2.3	4.8	1.0	0.5	1.9	3.5	0.02	0.01	0.02	0.05
*Persea americana*	Lauraceae	3.7	2.9	2.8	9.4	4.5	3.7	2.9	11.1	-	-	-	-
*Psidium guajava*	Myrtaceae	5.0	6.8	4.9	16.8	4.4	5.1	4.2	13.7	-	-	-	-
*Rhamnus prinoides*	Rhamnaceae	4.7	4.7	3.5	12.9	4.6	5.3	4.2	14.1	0.048	0.046	0.045	0.14
*Rhus glutinosa*	Anacardiaceae	1.0	0.6	2.2	3.8	1.2	0.6	1.7	3.5	0.03	0.03	0.04	0.10
*Schinus molle*	Anacardiaceae	0.8	0.5	1.4	2.6	1.4	0.8	2.0	4.2	0.02	0.015	0.02	0.055
*Sesbania sesban*	Fabaceae	3.3	2.4	2.5	8.3	-	-	-	-	0.07	0.07	0.04	0.18
*Ziziphus spina-christi*	Rhamnaceae	5.6	7.7	4.6	17.9	5.3	8.7	5.6	19.6	0.02	0.01	0.04	0.07

LE, Lower Elevation; ME, Middle Elevation; HE, Higher Elevation; RF, Relative Frequency; RD, Relative Density; RDo, Relative Dominancy; IVI, Important Value Index.

### 3.3 Farmers’ perception of the integration of tree/shrub plantations to the agricultural lands

**Figure 4:**
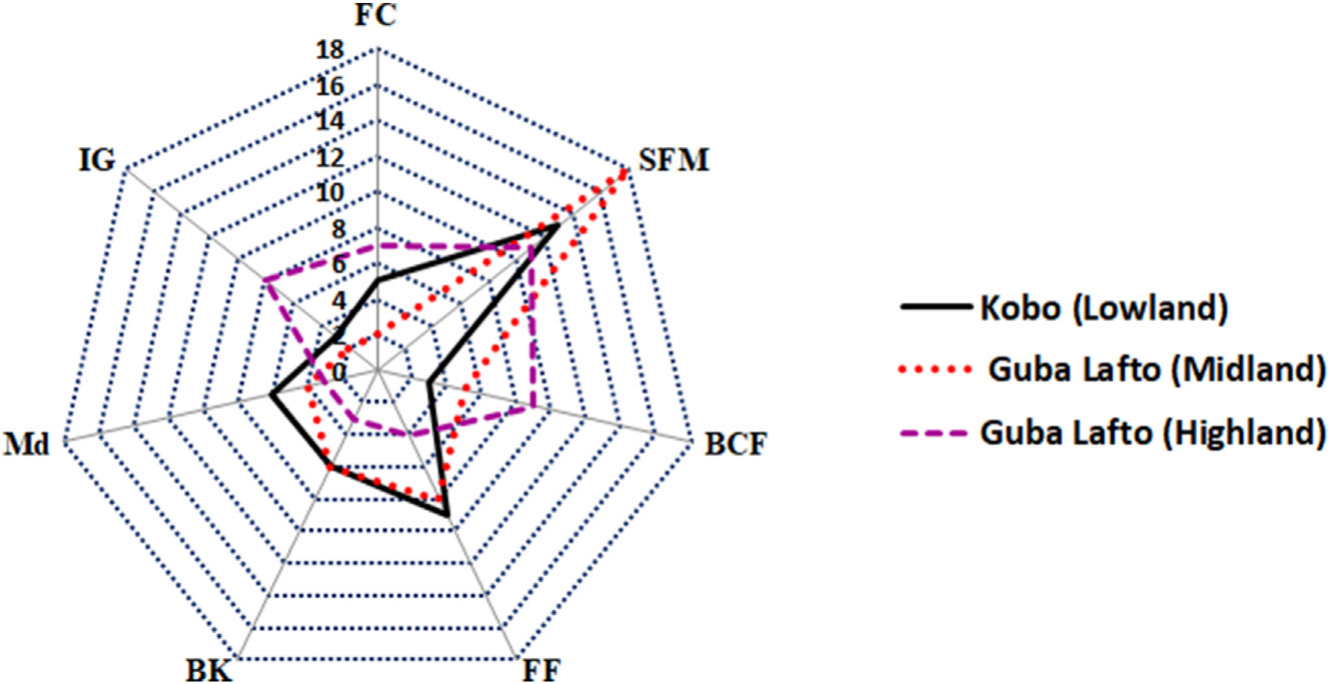
Radar chart illustrating farmers' perception on benefits of integrating trees on agricultural lands across an elevation gradient in North-eastern Ethiopia. Key: FC, Fuel, and Charcoals; SFM, Soil Fertility Maintenance; BCF, Building Construction, and Fence; FF, Food and Fodder; BK, Bee Keeping; Md, Medicinal use; IG, Income Generation.

Among the total respondents (135), 106 (78.5%) farmers integrated trees/shrubs into their agricultural systems. Such knowledge of farm tree plantation was acquired from their parents (i.e., an indigenous knowledge transfer) accounting for 48 (45.3 %), through observation and training from developmental associations 31 farmers (29.2%) and non-governmental organizations 21 farmers (19.8 %). Six (5.7%) of the respondents unveiled that tree species such as *Z. spina-christi* and *A. seyal* are often dispersed by birds (locally called: *wofe zerash*) on their farmlands without farmers' involvement. Regarding tree plantation practices and distribution, the highest tree density was reported around homesteads (51.1%), followed by farm boundary (30.4%), and in the-farm lands (13.3%) whilst few (5.2 %) respondents prefer to plant a tree on pastoral and degraded lands. The purpose of tree plantation in on-farm systems is indicated in Figure 4. Out of the 135 respondents, 42 (31.5 %) practiced tree plantation in their farmlands for soil fertility maintenance, followed by 21 for food and fodder (15.5 %), and 17 for building, construction, and fence (12.5 %). As indicated in Table 5, *Z. spina-christi* was found to be the most preferred tree species for soil fertility maintenance, followed by *Cordia africana* and *Ficus vasta*. However, the lowest ranks were given to *Ehretia cymosa* and *Acacia senegal*. Notwithstanding the belief that trees are necessary, farmers in the districts described the distribution of multi-purpose native farm tree species (e.g., *Acacia abyssinica*, *Hagenia abyssinica*, *Podocarpus falcatus*, etc) are gradually declining in the agricultural systems and replaced by some exotic species such as Eucalyptus, which depreciates the soil fertility potential of the agricultural lands. In the study area, farmers’ preference of tree/shrub species to maintain the soil fertility status is varied.

**Table 5:** Respondents' (R1-R10; “key informants”) preference ranking for ten selected tree species based on the assumed maintenance of soil fertility. The rank was determined following the grading of ten most planted tree species to boost soil fertility; the largest value (10) was assigned to species considered to hold the greatest importance for soil maintenance, while the least contribution to soil maintenance was assigned (1).

Tree species	R1	R2	R3	R4	R5	R6	R7	R8	R9	R10	Total	Rank
*Acacia senegal* ​(L.) ​Willd.	1	1	2	2	1	1	2	1	1	1	13	10
*Acacia seyal* ​Delile	4	4	3	4	5	4	4	5	5	3	41	7
*Albizia schimperiana* ​Oliv.	3	3	4	3	2	3	3	3	2	4	30	8
*Cordia africana* ​Lam.	8	9	10	8	8	9	10	8	10	8	88	2
*Cordia myxa* ​Thwaites	6	6	5	6	6	6	7	4	7	5	58	5
*Croton macrostachyus* ​Hochst. ​ex ​Delile	7	7	8	7	9	7	6	9	6	7	73	4
*Ehretia cymosa* ​Willd. ​ex ​Roem. ​& ​Schult.	2	2	1	1	3	2	1	2	3	2	19	9
*Ficus vasta* ​Forssk.	9	8	7	9	7	10	8	7	9	9	83	3
*Olea europaea* ​subspec. ​*cuspidata* ​(Wall. & G.Don) Cif.	5	5	6	5	4	5	5	6	4	6	51	6
*Ziziphus spina-christi* ​(L.) ​Willd.	10	10	9	10	10	8	9	10	8	10	94	1

## 4 Discussion

### 4.1 Composition of tree/shrub plantations across an altitudinal gradient and on-farm niches

The density and distribution of tree species in the farmlands are influenced by various unified factors, including topography, biophysical attributes, and socio-economic conditions (Nogues-Bravo et al., 2008; Negash et al., 2012; Dhakal et al., 2012; Haile et al., 2017; Sharma et al., 2017). Among these, elevation and tree niches have served as important determinant factors for tree plantation and distribution of trees in the agricultural systems (Nogues-Bravo et al., 2008; Haile et al., 2017). In this study, monotonic decreases in stem density and taxon were observed from lower to higher elevation gradients. A similar decrease in numbers of tree species with an increase in elevation could result from poor soil nutrient concentration and organic matter decomposition at the higher altitudinal gradients, caused by lower temperature and higher precipitation (Duguma and Hager, 2010; Negash et al., 2012; Haile et al., 2017; Monge-Gonzalem et al., 2019). In terms of tree niches, the homesteads had a higher number of taxa and stem density in the entire altitudinal gradient as compared with the boundary and on-farm plantations (Table 5). This was in line with other similar studies in Ethiopia (e.g., Abebe, 2005; Tolera et al., 2008; Duguma and Hager, 2010), which described a marked increase in numbers of taxa, stem, and species diversity in the home gardens than in the other land-use types. These studies also showed variations in species heterogeneity with changes in elevation gradients and plantation niches. The reduction in tree species diversity along with elevations (from lower to higher gradients) (Figure 3), could be ascribed to environmental variability in terms of soil characteristics, temperature, species adaptability, and management practices. Similar trends of decreasing tree species diversity with altitudinal gradients have been reported in Southern Ethiopia (Tolera et al., 2008), South-eastern Ethiopia (Negash et al., 2012), and central Ethiopia (Haile et al., 2017). The present study showed that *A*. *seyal*, *Z. spina-christi*, *P. guajava*, and *M. indica* contributed the highest IVI in the lower and middle elevations, while *E. globulus* in the higher altitudinal gradient (Table 4). The highest IVI of these species might be associated with their socioeconomic values (e.g., construction wood, firewood, and income generation), ecological significance (e.g., soil fertility maintenance, shade, e.t.c), and greater ecological success (e.g., *A. seyal,* and *Z. spina-christi*) (Table 4). Similar observations of higher IVI for *Z. spina-christi* in the home-garden agroforestry systems in Northern Ethiopia and *C. arabica,* P. americana, and *M. indica* in the home garden and parkland in Southern Ethiopia have been reported by Eyasu et al. (2020) and Legesse and Negash (2021), respectively. Other studies conducted by Tolera et al. (2008), Kassa et al. (2015), and Molla and Kewessa (2015) showed that *Acacia falcata*, *Croton macrostachyus*, *Ficus sur* Forssk., and *Eucalyptus camaldulensis* were the top important woody species in different land use types, which could be due to their economic role and ecological requirement of the life strategy of the species.

### 4.2 Farmers’ perception to integrate trees on the farm systems

Trees on agricultural lands play a vital role to boost agricultural productivity and resilience of smallholder's farming system from conservation of biological diversity to provision of essential ecosystem services (Kebebew and Urgessa, 2011; Cerdan et al., 2012; Dhakal et al., 2012; Abebe et al., 2013; Jackson et al., 2013). Thus, the majority of the surveyed farmers integrated trees into their farm system to improve soil fertility, get farm utilities, and livestock food and fodder. The perception of farmers on tree plantations in the present study coincides with other studies that highlight the importance of farm plantations on agricultural systems in different areas (McNeely and Schroth, 2006 and Cerdan et al., 2012). Besides, Asfaw and Agren (2007) showed the relevance of indigenous AF systems dealt with the management of soil fertility. Different studies (Soto-Pinto et al., 2007; Dhakal et al., 2012; Hasan et al., 2014; Amare et al., 2019), on their part, described that integrating trees in farmlands provide a variety of benefits, including livelihoods, ecosystem services, and the existence of scenic places. However, as reported by interviewed farmers status of indigenous tree species on the farm system in north Ethiopia is declining from time to time. The decreasing trends of AF practices year to year resulted from the reckless cutting of trees for charcoal, construction, timber, and farm implements as reported by Alebachew (2012) in western Shewa Zone of central Ethiopia. On the other hand, farmers asserted comparatively higher tree/shrub diversity around their homesteads and boundary than in their farmlands. The lowest tree diversity in farmlands could be associated with farm trees resource completion, such as soil moisture and nutrients with the adjacent cultivated crops (Harrison et al., 2000; Alebachew, 2012). On the other hand, as reported by farmers, they prefer to grow tree species such as *Z. spina-christi*, *Cordia africana* (Lam), and *Ficus vasta* as these tree species are effective in improving the soil fertility status of their farmlands (Table 5). The result concurs with Gindaba et al. (2005) that reported the practice and wide preference of *C. africana*, *F. vasta*, and *C. macrostachyus* tree plantations in cultivated lands by the farmers of Badessa areas in eastern Ethiopia. The authors confirmed that soil available phosphorus under these tree species canopies was (34–50 %) higher than the corresponding soil away from the canopies. The integration of *C. africana* and *Millettia ferruginea* for soil fertility maintenances has also been reported in some locations of Ethiopia. For instance, Asfaw and Agren (2007) reported a significant improvement in the concentration of available phosphorus under the canopies of *Cordia africana* Lam and *Millettia ferruginea* Hochst in Sidama, southern Ethiopia. However, other tree species, such as *Eucalyptus camaldulensis, Eucalyptus grandis*, and *Eucalyptus pellita* depleted soil resources, compete with other plants for soil moisture and nutrients, and have a significant negative effect on soil physicochemical properties (Harrison et al., 2000).

## 5 Conclusions

Agroforestry is a complex ecological-based natural resource management system with many benefits. Farmers in north Ethiopia have been integrating shrubs and trees into their agricultural systems to assure sustainability and productivity. However, tree species density and variability declined with increasing altitudinal gradient and many multipurpose tree species are drastically deteriorated on-farm systems and replaced by high adaptive exotic tree species due to their socioeconomic importance for fuel, construction, fence, food, and ease to propagation. But, these exploit more resources including water from the soil. Therefore, farmers and agroforestry expertise should emphasize composing their plantations with the species ideal for soil fertility maintenance, such as *A. abyssinica*, *H. abyssinica*, and *P. falcatus*, higher IVI, and tree species with higher preferences to the farmers for soil fertility improvement. To this end, the study showed that tree species diversity and composition is shifting to non-native trees. This calls for designing management options, such as the establishment of native tree species nursery, identification of multifunctional and keystone tree species, and make suitability analysis that respond to various physiographic condition by formulating the farmers' indigenous tree plantation practices. Furthermore, more comprehensive studies are needed to investigate the short and long-term benefits of agroforestry systems in the provision of ecosystem services.

## Declarations

### Author contribution statement

Meseret Muche: Conceived and designed the experiments; Performed the experiments; Analyzed and interpreted the data; Wrote the paper.

Eyayu Molla; Boris Rewald: Conceived and designed the experiments; Analyzed and interpreted the data; Wrote the paper.

Berhanu Abraha Tsegay: Conceived and designed the experiments; Wrote the paper.

### Funding statement

This research did not receive any specific grant from funding agencies in the public, commercial, or not-for-profit sectors.

### Data availability statement

Data included in article/supplementary material/referenced in article.

### Declaration of interests statement

The authors declare no conflict of interest.

### Additional information

No additional information is available for this paper.
