# Supplementary material for: Diversity and composition of farm plantation tree/shrub species along altitudinal gradients in North-eastern Ethiopia: implication for conservation
Source: Heliyon. 2022 Mar 5;8(3):e09048. doi: 10.1016/j.heliyon.2022.e09048 (PMC8917278; doi:10.1016/j.heliyon.2022.e09048)
Supplement: Supplementary [file mmc1.doc]

**Supplementary 1.** Description of the study Districts (n=2) and Sites (n=9) based on their altitudinal gradients and corresponding agro-climatic conditions.

| **Districts** | **Surveyed Sites** | **Elevation** | **Agroclimate** | **Coordinates** | |
| --- | --- | --- | --- | --- | --- |
|  |  | **Gradients** | Zones | X | Y |
| Kobo | Kobo zuria | Lowland | Hot | 12.15405N | 39.633856E |
|  | Robbit | Lowland | Hot | 12.124895N | 39.637758E |
|  | Behoro/Gobye | Lowland | Hot | 11.979850N | 39.648745E |
| Guba Lafto | Gedober/Jarsa | Midland | Temperate | 11.883109N | 39.525149E |
|  | Anova | Midland | Temperate | 11.885796N | 39.514162E |
|  | Woyeneye | Midland | Temperate | 11.901922N | 39.472963E |
|  | Geshober | Highland | Cool | 11.904610N | 39.405498E |
|  | Debo/Sikela | Highland | Cool | 11.944919N | 39.423525E |
|  | Bequlo maneqiya /Dilb | Highland | Cool | 11.987910N | 39.426272E |

**Supplementary 2.** The questionnaire survey on the farmers’ perception of tree plantation practices in their locality and its implication for maintaining soil fertility.

**Dear respondents,**

This questionnaire is a research tools for the “**Effects of Altitudinal Gradient on the Diversity and Composition of Farm Plantation Tree Species and Implications for Soil fertility: Exploration in Northeastern Ethiopia**” to gather information on the farmers perception of the implications of tree plantations for soil fertility maintenances. The questionnaires are divided into three segments, which are the HH characteristics, exploration of the farmers' tree plantation practices and intervention mechanisms, and assessments of the farmers' perception of tree plantation for soil quality improvements. Additionally, the questionnaire has the following objectives:

- To explore the major tree/shrub plantation by the farmers on the farmland systems.
- To appraise the farmers' perception of planting a tree for soil fertility maintenance and adoption mechanisms.
- To provide suggestions on the future conservation and maintenances of soil fertility.

Therefore, I request your honest and valuable responses to fill up this questionnaire. Your candid response is highly relevant to the study.

**Part One: the demographic characteristics of the surveyed respondents**

Date of interview________________

Survey area: Region _____ District __________ Study site _________ Code _______

Age _______ Sex: Male Female

Marital status: Married Single Divorced Other Family size: __________

Academic status: no formal schoolings primary schoolings

Secondary Education tertiary schoolings

**Part TWO: Questionnaire related to tree plantation on farmlands**

1. Do you think tree plantations (AF) are practiced in your locality?

Yes No I don’t realize

1. Have you planted trees/shrubs with the integration of agricultural lands?

Yes No I don’t realize

1. If your answer for question no. 2 is ‘Yes’, which tree/shrub species is/are probably integrated to which land uses types and crop/s?

| No | Tree/shrub | Land use types | The more likely integrated Crop types | Quality of the soil and crops after integration: High; Medium and Low |
| --- | --- | --- | --- | --- |
|  |  |  |  |  |
|  |  |  |  |  |
|  |  |  |  |  |
|  |  |  |  |  |
|  |  |  |  |  |
|  |  |  |  |  |
|  |  |  |  |  |

1. In which sites of the agricultural land use types do you practice the tree plantation?

Boundary Home Stead Center of farmland

Degraded Lands Pasture land Others ___________

1. How do you rate the density of tree plantations in your agricultural plots and locality in general?

High Medium Low Very Low

1. If your answer for Q5 is rated low, what factors are attributed to the reduction of tree plantation practices in your locality?

__________________________________________________________________________________________________________________________________________________________

1. If your answer for Q5 is high, where did you get the experience or adoption strategies?

Indigenous Knowledge (IK)

Non-governmental organization (NGO)

Developmental Association (DA)

Others

1. What do you think about the status of native tree planation practice in your locality? Decreasing or Increasing trends _______________________________________,
2. What factors and threats attribute for increasing or decreasing trends? ____________________

**Part Three: Tree plantations for soil fertility maintenances**

1. Do you think AF practices improve the soil fertility and productivity status of your farmlands compared to the non-agroforestry farmlands system?

Yes No I don’t realize

1. What is/ are the implication/s and main preferences for planting a tree on your farmlands? For:

Fire wood/ Charcoal Soil fertility maintenance

Building/construction and Fence Food and Fodder Bee Keeping Medicine Income generation others _____________________

1. What possible solution would be implemented to improve the agroforestry practices in your locality? ________________________________________________________________________________________________________________________________________________________________

**Supplementary 3.** The number/s and its proportion of tree/shrub species in each of the family.

|  | **Frequency** | **Percent (%)** |
| --- | --- | --- |
| Acanthaceae | 1 | 2.7 |
| Apocynaceae | 1 | 2.7 |
| Caricaceae | 1 | 2.7 |
| Celactraceae | 1 | 2.7 |
| Ebenaceae | 1 | 2.7 |
| Lauraceae | 1 | 2.7 |
| Moraceae | 1 | 2.7 |
| Moringaceae | 1 | 2.7 |
| Oleaceae | 1 | 2.7 |
| Proteaceae | 1 | 2.7 |
| Rosaceae | 1 | 2.7 |
| Rubiaceae | 1 | 2.7 |
| Sapindaceae | 1 | 2.7 |
| Rhamnaceae | 2 | 5.5 |
| Myrtaceae | 3 | 8.1 |
| Rutaceae | 3 | 8.1 |
| Boraginaceae | 3 | 8.1 |
| Euphorbiaceae | 3 | 8.1 |
| Anacardiaceae | 3 | 8.1 |
| Fabaceae | 7 | 18.9 |
|  | 37 | 100 |
